# A Case of Intra-abdominal Hemorrhage Following a Failed Kristeller Maneuver With Vacuum-Assisted Delivery

**DOI:** 10.7759/cureus.116829

**Published:** 2026-09-24

**Authors:** Tetsu Yamaguchi, Shunsuke Tamaru, Namba Akira, Takeshi Kajihara, Yoshimasa Kamei

**Affiliations:** 1 Department of Obstetrics and Gynecology, Saitama Medical University, Saitama, JPN

**Keywords:** abnormal bleeding during childbirth, hysterectomy, intra-abdominal hemorrhage, kristeller maneuver, vacuum-assisted-delivery

## Abstract

We report a case of intra-abdominal hemorrhage associated with vascular injury in the recto-vaginal space and peritoneal injury in the Douglas pouch following vacuum-assisted delivery with an unsuccessful Kristeller maneuver. The patient was 41 years old, gravida 2, para 1. At 40 weeks gestation, labor was induced by administering oxytocin. Due to prolonged second stage of labor in breech presentation, vacuum-assisted delivery with the Kristeller maneuver was attempted in vain, followed by forceps delivery, resulting in the delivery of a female infant weighing 3,178 g. However, at two hours postpartum, the patient went into a state of shock, with a blood pressure of 59/41 mmHg and pulse rate of 98 beats/minute, and was transferred to our tertiary care center. Exploratory laparotomy revealed bleeding from peritoneal injury in the Douglas pouch, serosal injury of the rectum, and also from the vaginal venous plexus at the site of connective tissue injury in the recto-vaginal space. There were no other sources of bleeding, including no uterine rupture in the birth canal. We hypothesized that a shearing effect around the recto-vaginal space and the Douglas pouch, possibly generated by the forces associated with instrumental delivery and the Kristeller maneuver, may have contributed to the connective tissue injury in the retroperitoneal space and peritoneal injury in the Douglas pouch. Although no direct evidence of uterine or vaginal injury was found, it is important to recognize the potential for injuries to the retroperitoneal space and abdominal cavity when performing instrumental delivery along with the Kristeller maneuver.

## Introduction

The Kristeller maneuver, introduced by Samuel Kristeller in 1867, is used to enhance expulsion force during the second stage of labor by applying pressure to the uterine fundus [1]. The Kristeller maneuver is known for its simplicity and effectiveness and is sometimes used in conjunction with instrumental delivery. However, the use of fundal pressure remains controversial, and the World Health Organization does not recommend its use to facilitate childbirth during the second stage of labor because of concerns regarding potential maternal and neonatal harm [2].

Cases of uterine rupture have previously been reported as some serious complications associated with vacuum-assisted delivery using the Kristeller maneuver [3]. However, cases of damage to retroperitoneal and intra-abdominal tissues at the pelvic level have not been reported to date. We hereby report a case of retroperitoneal and intra-abdominal tissue damage at the pelvic level with no incidence of uterine, cervical, or upper vaginal wall lacerations after an unsuccessful attempt at instrumental extraction assisted by the Kristeller maneuver.

## Case presentation

The patient was a 41-year-old woman, gravida 2, para 1, with a height of 160 cm and a weight of 55 kg at delivery (body mass index (BMI): 21.5 kg/m²). Her previous pregnancy resulted in an uncomplicated vaginal delivery at 38 weeks of gestation of a female infant weighing 2,938 g. She had no history of uterine aspiration or uterine surgery, including cesarean delivery. This pregnancy was managed at a primary care facility, with no significant abnormalities noted during the course of gestation. Her pre-delivery hemoglobin level was 11.6 g/dL.

At the gestation period of 40 weeks and five days, the induction of labor with oxytocin infusion was initiated. The patient showed spontaneous rupture of membranes, and complete uterine cervical dilation was achieved at 90 minutes after the onset of labor. Two hours into the second stage of labor, the fetus showed a bregmatic presentation with the fetal head at station +4, and instrumental delivery was attempted due to the indication of arrest of labor. Two attempts at vacuum extraction using a soft cup in the axis of the birth canal at 50 mmHg with the Kristeller maneuver were applied in vain; the Kristeller maneuver was performed by a midwife. The soft cup slipped off quickly, with a traction time lasting approximately 2 seconds per attempt. During this maneuver, the patient complained of pain extending from the pelvis to the buttocks during the second vacuum extraction attempt. Subsequently, forceps delivery was performed, and a 3178 g female infant with an Apgar score of 9 at one minute and 10 at five minutes, an umbilical artery pH of 7.24, and a base excess of -5.9 was delivered. The intrapartum cardiotocographic tracing during these procedures is shown in Figure 1.

**Figure 1:**
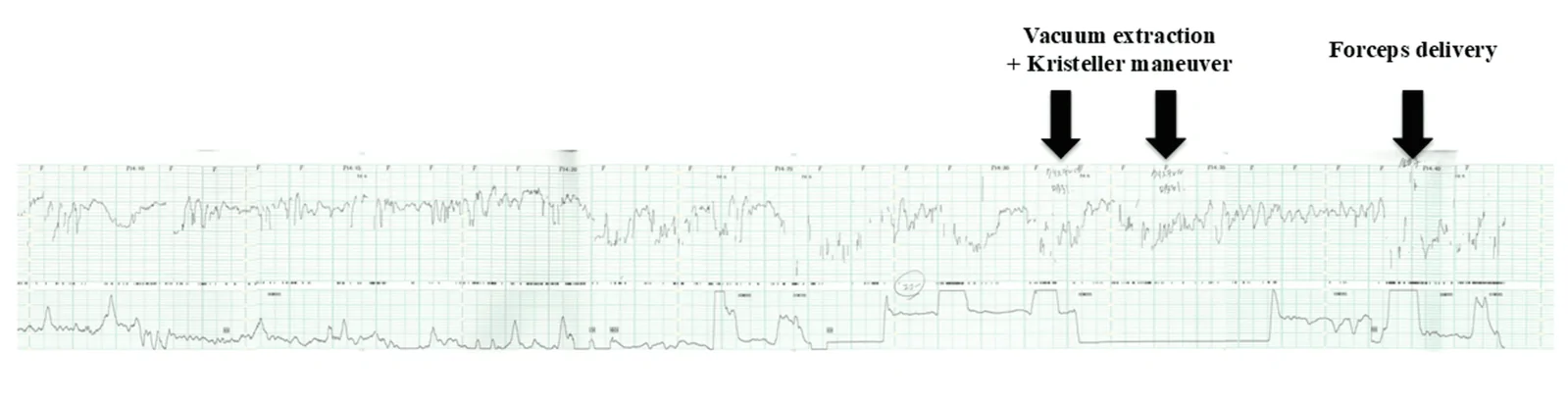
Intrapartum cardiotocographic tracing during operative vaginal delivery The arrows indicate the two attempts at vacuum extraction with the Kristeller maneuver and the subsequent forceps delivery.

The placenta was delivered spontaneously five minutes after birth. Total blood loss at this point was approximately 310 mL.

At one hour postpartum, the patient was alert and oriented with a blood pressure of 128/87 mmHg, a pulse rate of 112 beats per minute (bpm), and approximately 60 mL of external bleeding but complained of severe pain in the buttocks. One hour postpartum, the patient showed a decreased level of consciousness in spite of low external blood loss of approximately 20 mL; her blood pressure dropped to 59/41 mmHg, pulse rate was 98 bpm, and the Shock Index score was 1.6 [4]. Transabdominal ultrasound revealed an echo-free space in the abdominal cavity, suspicious of intra-abdominal hemorrhage, and the patient was transferred to our hospital, which is a tertiary care facility.

Four hours postpartum, she was transferred with a Glasgow Coma Scale score of 12, blood pressure of 69/40 mmHg, and a pulse rate of 110 bpm (Shock Index 1.6) [4]. The blood tests are as follows: hemoglobin 6.2 g/dL, platelet count 174×109/L, fibrinogen 354 mg/dL, and d-dimer 13.8 μg/mL. Coagulation tests showed an activated partial thromboplastin time of 33.2 seconds, a prothrombin time of 8.4 seconds, and a prothrombin time-international normalized ratio of 0.86. Blood gas analysis showed a pH of 7.268, a base excess of −10.8 mEq/L, and a lactate level of 3.8 mmol/L. The patient had several perineal/vaginal lacerations with no signs of hematoma. Transabdominal ultrasound revealed neither retained placental tissue nor uterine muscle layer rupture. Instead, an echo-free space extending into the Morrison’s pouch was identified, suggesting intra-abdominal hemorrhage, and emergency laparotomy was performed.

At laparotomy, the amount of intra-abdominal blood with clotting was approximately 3000 ml. The uterus itself was intact; however, persistent bleeding was noted near the Douglas pouch (Figure 2), and bleeding was confined to the pelvic cavity.

**Figure 2:**
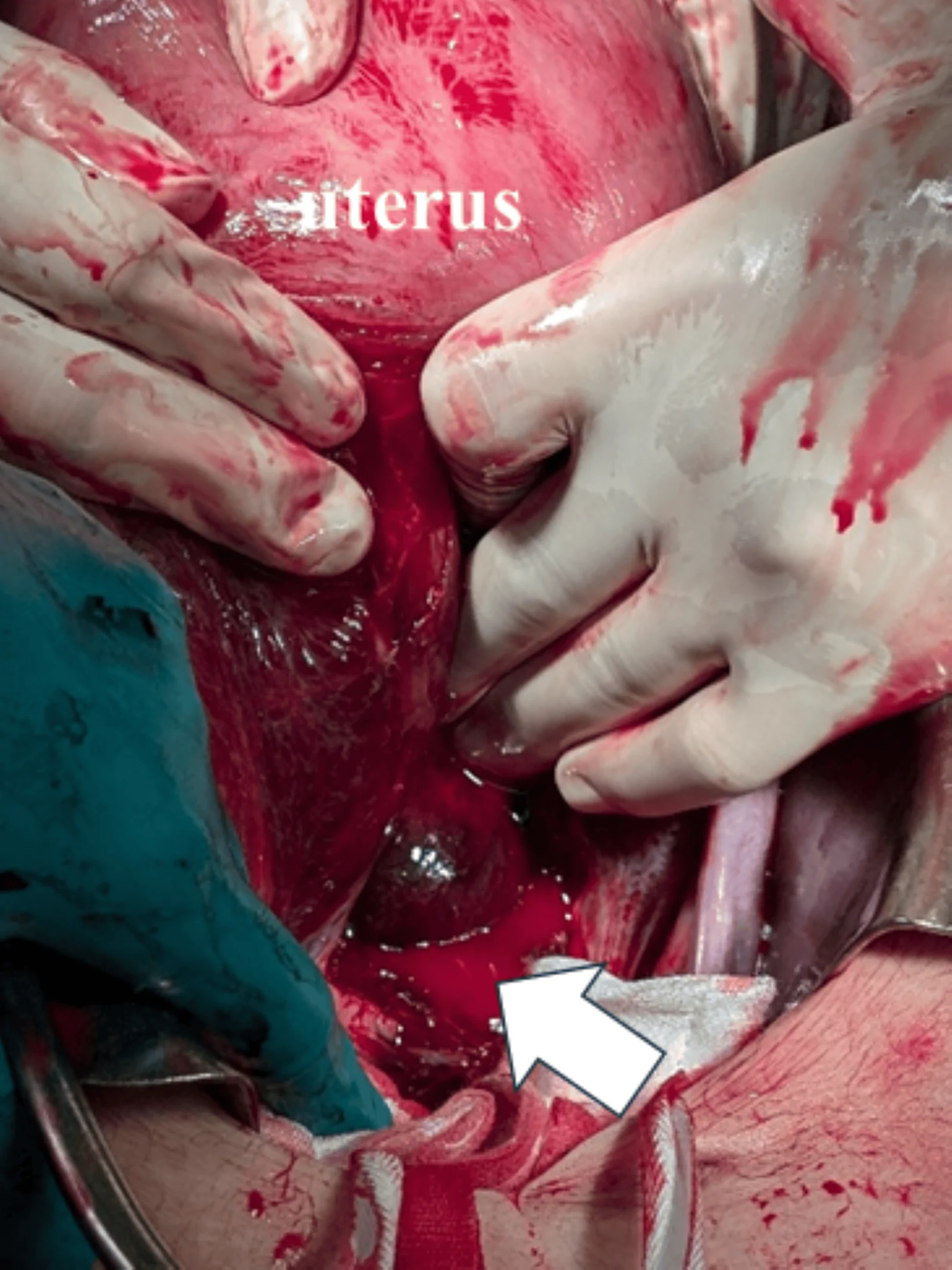
Pelvic photograph before hysterectomy Bleeding was detected near the Douglas pouch (arrow).

Identification of the bleeding source at the Douglas pouch was difficult because of the large postpartum uterus, and a total hysterectomy was performed prior to hemostasis. Extensive damage in the recto-vaginal space, peritoneal injury in the Douglas pouch, and serosal injury of the rectum were confirmed following uterine removal (Figure 3), and the bleeding from the vaginal venous plexus persisted, necessitating the use of a sheet-like biological tissue adhesive/sealant in addition to ligation and coagulation hemostasis.

**Figure 3:**
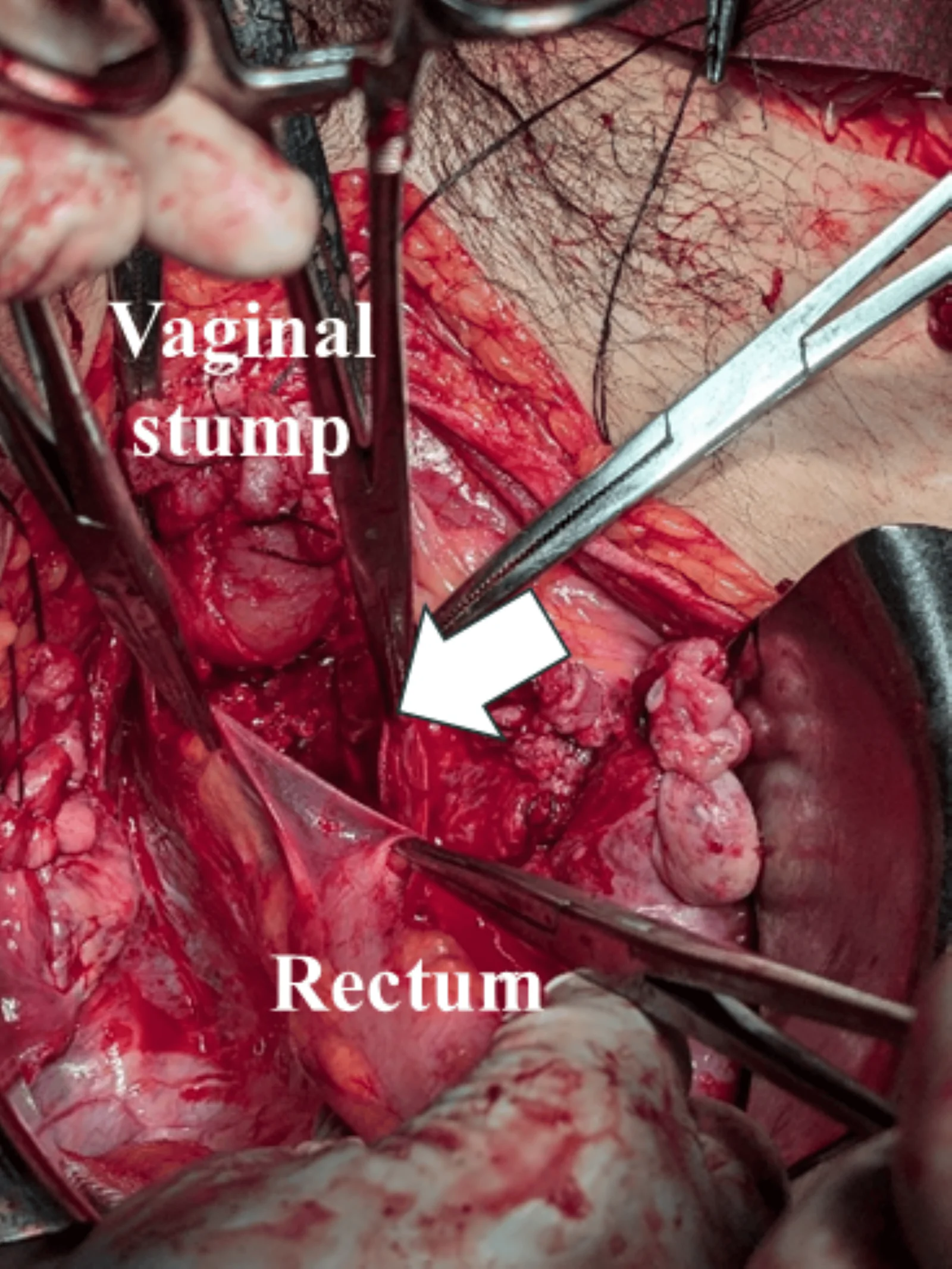
Pelvic photograph after hysterectomy Severe damage to the serosa of the rectum and surrounding connective tissue was noted (arrow).

Intraoperative total blood loss was 6030 mL, requiring massive transfusion therapy consisting of 20 Japanese units (2,800 mL) of red blood cell concentrate, 16 Japanese units (1,920 mL) of fresh frozen plasma, 20 Japanese units (400 mL) of platelet concentrate, and 3 g of human fibrinogen concentrate. The postoperative hemoglobin level was 7.4 g/dL. 

No gross damage was observed on the excised uterus (Figure 4).

**Figure 4:**
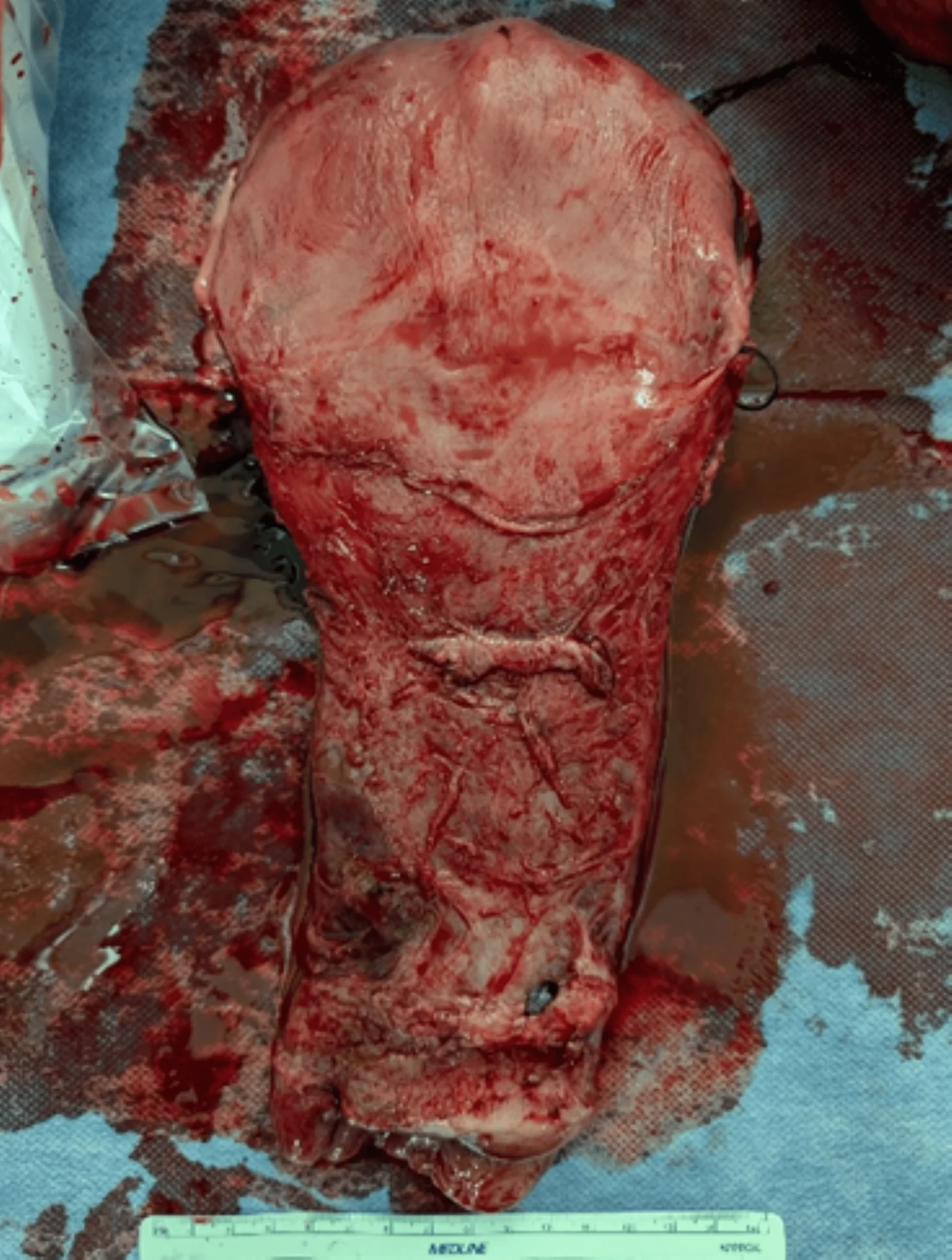
Photograph of the front surface of the excised uterus No gross damage was observed.

The pathological diagnosis was consistent with this finding. The postoperative course was uneventful, and on the fifth postoperative day, the patient was referred to the original obstetric clinic.

## Discussion

The Kristeller maneuver technique has been associated with enhanced risk of complications such as uterine rupture, bladder rupture, uterine inversion, and third- and fourth-degree lacerations [5] and has been considered contraindicated in some countries, primarily in Europe [6]. On the contrary, reports of maternal complications during vacuum extraction are rare, with the most common being anal sphincter injuries [7]. In rare cases, uterine rupture has been reported, with risk factors of the procedure being incomplete uterine cervical dilation, cephalopelvic disproportion, scarred uterus, and prolonged traction [8]. However, cases involving the retroperitoneal cavity without uterine or birth canal injury during instrumental delivery assisted by the Kristeller maneuver have not been reported.

In this case, the intra-abdominal bleeding may have occurred during the second vacuum extraction assisted by the Kristeller maneuver, when the patient experienced severe pain in the buttocks. When vacuum-assisted delivery was attempted, more than two hours had already passed since full uterine cervical dilation, and the fetal head was at station +4, which satisfied the full indications and summary for vacuum-assisted delivery [9], but in vain because of the malrotation of the fetal head. In the present case, after the initial vacuum attempt was unsuccessful in the presence of fetal head malrotation, the feasibility and safety of further operative vaginal delivery should have been carefully reassessed before repeating the procedure. We hypothesize that under these circumstances, the force was applied in the upper posterior vagina and fornix and may have generated shear forces to the peritoneum and connective tissue surrounding the Douglas pouch. However, the precise mechanism of injury cannot be definitively established from this single case.

Taking into account the Kristeller maneuver technique, which originally involved placing both palms on the fundus of the uterus to massage it, as well as repeatedly applying pressure in the longitudinal direction of the birth canal for a short period [1], the external force applied and the site of injury at the time of damage have been depicted in Figure 5.

**Figure 5:**
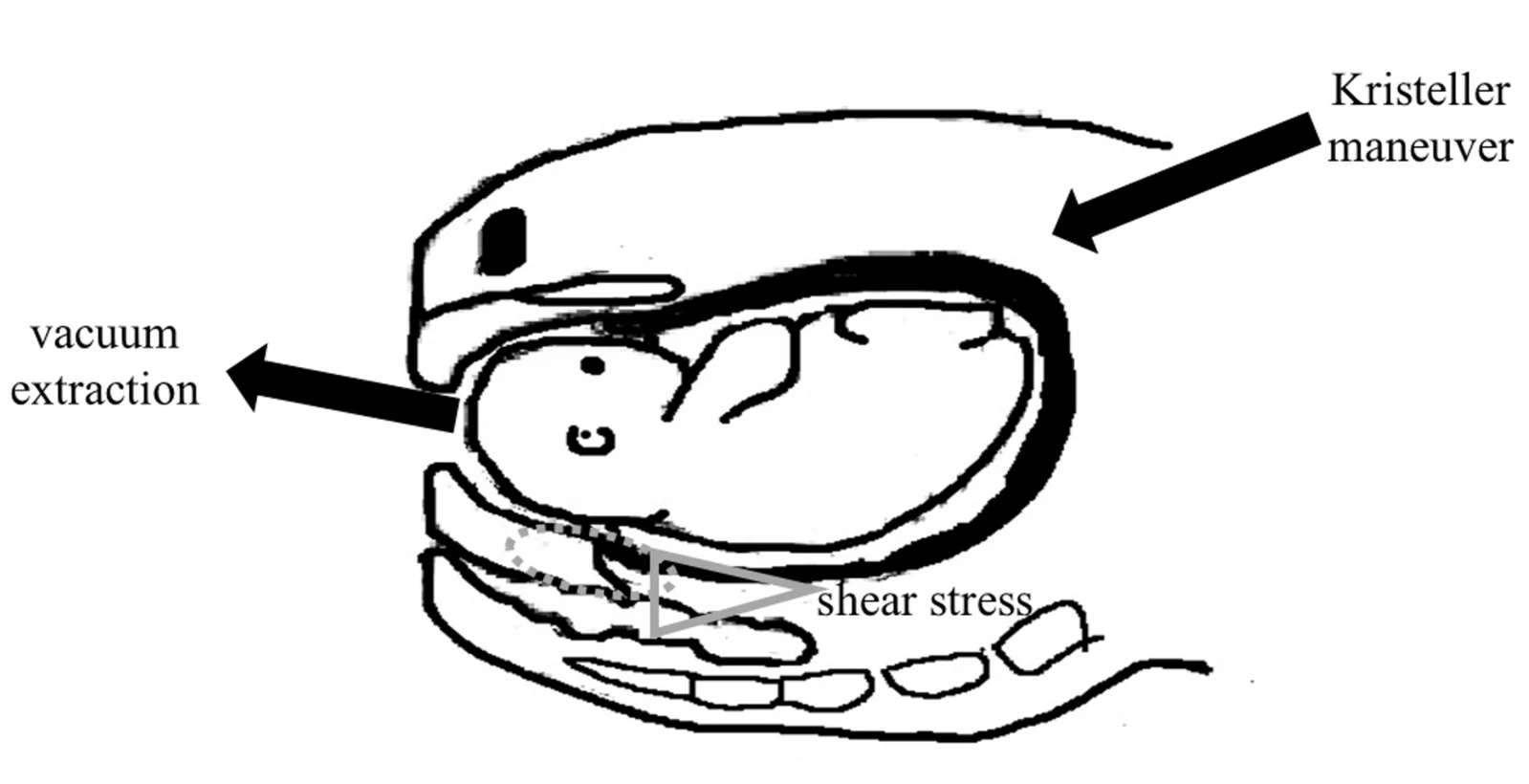
Schematic diagram of the pelvic area The illustration shows the hypothesized mechanism of injury. The black arrows represent the directions of applied forces by vacuum extraction and the Kristeller maneuver. These forces are assumed to have generated shear stress in the direction of the gray arrowhead in the tissues near the Douglas pouch. The damaged section is represented by the gray dotted circle. Image credit: The illustration was hand-drawn by the authors, scanned, and digitally edited using Microsoft Paint (Microsoft Corporation, Redmond, WA, USA).

Although it might be argued that this injury is just because of the involvement of the vaginal wall or uterine cervix at the time of application of the vacuum cup, it is unlikely because the cervix was fully dilated and the fetal head was descended enough to Station +4, and there were no large cervical/vaginal lacerations and hematoma. It is also unlikely that forceps delivery was involved in this massive intra-abdominal bleeding because the onset of severe pain before forceps delivery suggests that the injury may have occurred before forceps application [10]. It has also been reported to be a risk factor for retroperitoneal hematoma [11]; however, massive intra-abdominal bleeding has never been reported, and this does not align with the course of this case. Furthermore, causes leading to intra-abdominal hemorrhage during or immediately post-delivery, which are not associated with the delivery techniques, include uterine rupture during pregnancy [12] as well as non-traumatic idiopathic intra-abdominal hemorrhage caused by endometriotic lesions [13], rupture of the ovarian artery [14], and rupture of a splenic artery aneurysm [15]; but in this case there were no endometriotic lesions within the abdomen and no rupture of major intra-abdominal arteries. Uterine rupture associated with placenta accreta spectrum, even in an unscarred uterus, should also be considered as a rare cause of intra-abdominal hemorrhage during pregnancy or the peripartum period [16]. However, no uterine rupture or findings suggestive of PAS were identified in the present case.

## Conclusions

Particular caution may be warranted when fundal pressure is used in conjunction with operative vaginal delivery. When an initial attempt at operative vaginal delivery is unsuccessful, particularly in the presence of malrotation or failure of descent, the likelihood of successful vaginal delivery should be reassessed before further attempts. Repeated operative vaginal delivery combined with fundal pressure should be avoided, and timely conversion to cesarean delivery should be considered when safe vaginal delivery is unlikely to be achieved. Furthermore, careful monitoring of the maternal condition is important after operative vaginal delivery combined with fundal pressure, particularly when severe pain or hemodynamic deterioration occurs despite limited external bleeding. 

## References

[1] Waszyński E (2008). Kristeller's procedure-expressio fetus, its genesis and contemporary application (Article in Polish). Ginekol Pol.

[2] World Health Organization (2018). World Health Organization: WHO Recommendations: Intrapartum Care for a Positive Childbirth Experience. WHO Recommendations: Intrapartum Care for a Positive Childbirth Experience.

[3] Masaoka R, Ohno M, Takamizu A, Koizumi A, Yoshida K, Makino S (2022). A case of unscarred uterine rupture complicated by bladder rupture after vacuum extraction delivery. Hypertens Res Pregnancy.

[4] Allgöwer M, Burri C (1967). "Shock index" (Article in German). Dtsch Med Wochenschr.

[5] Hasegawa J, Sekizawa A, Ishiwata I, Ikeda T, Kinoshita K (2015). Uterine rupture after the uterine fundal pressure maneuver. J Perinat Med.

[6] Sagi-Dain L, Maymon R (2022). The condemned fundal pressure maneuver: time to reconsider?. Arch Gynecol Obstet.

[7] Abbas RA, Qadi YH, Bukhari R, Shams T (2021). Maternal and neonatal complications resulting from vacuum-assisted and normal vaginal deliveries. Cureus.

[8] Fahmy K (1976). Uterine rupture and vacuum extraction. Int J Gynaecol Obstet.

[9] Nishio E, Ishitani K, Arimoto T (2024). Guideline for gynecological practice in Japan: Japan Society of Obstetrics and Gynecology and Japan Association of Obstetricians and Gynecologists 2023 edition. J Obstet Gynaecol Res.

[10] Hossein-Pour P, Rajasingham M, Muraca GM (2025). Risk of cervical laceration in forceps vs vacuum delivery: a systematic review and meta-analysis. Acta Obstet Gynecol Scand.

[11] Vuong AD, Pham TH, Nguyen XT, Trinh NB, Nguyen PN, Ho QN (2023). Spontaneous hemoperitoneum in the second and third trimester of pregnancy: two uncommon case reports at Tu Du Hospital, in Vietnam and a literature review. Int J Emerg Med.

[12] Propst AM, Thorp JM Jr (1998). Traumatic vulvar hematomas: conservative versus surgical management. South Med J.

[13] Lier MC, Malik RF, Ket JC, Lambalk CB, Brosens IA, Mijatovic V (2017). Spontaneous hemoperitoneum in pregnancy (SHiP) and endometriosis - a systematic review of the recent literature. Eur J Obstet Gynecol Reprod Biol.

[14] Stanborough R, Hamrick K, Stephens C (2018). Spontaneous post-partum rupture of bilateral ovarian arteries treated with transarterial embolization. J Obstet Gynaecol Res.

[15] Ballout RA, Ghanem R, Nassar A, Hallal AH, Ghulmiyyah LM (2019). Splenic artery aneurysm (SAA) rupture in pregnancy: a case report of a rare but life-threatening obstetrical complication. J Womens Health Dev.

[16] Vuong AD, Nguyen XT, Nguyen PN (2022). Placenta accreta spectrum on an unscarred uterus in the third-trimester pregnancy: two rare cases at Tu Du Hospital in Vietnam. Int J Surg Case Rep.

